# The Impact of Neoadjuvant Hormone Therapy on Surgical and Oncological Outcomes for Patients With Prostate Cancer Before Radical Prostatectomy: A Systematic Review and Meta-Analysis

**DOI:** 10.3389/fonc.2020.615801

**Published:** 2021-02-08

**Authors:** Lijin Zhang, Hu Zhao, Bin Wu, Zhenlei Zha, Jun Yuan, Yejun Feng

**Affiliations:** Department of Urology, Affiliated Jiang-yin Hospital of the Southeast University Medical College, Jiang-yin, China

**Keywords:** prostate cancer, radical prostatectomy, neoadjuvant hormone therapy, meta-analysis, clinical research

## Abstract

**Objective:**

This systematic study aimed to assess and compare the comprehensive evidence regarding the impact of neoadjuvant hormone therapy (NHT) on surgical and oncological outcomes of patients with prostate cancer (PCa) before radical prostatectomy (RP).

**Methods:**

Literature searches were performed according to the Preferred Reporting Items for Systematic Reviews and Meta-Analyses guidelines. Using PubMed, Web of Science, Chinese National Knowledge Infrastructure, and Wanfang databases, we identified relevant studies published before July 2020. The pooled effect sizes were calculated in terms of the odds ratios (ORs)/standard mean differences (SMDs) with 95% confidence intervals (CIs) using the fixed or random-effects model.

**Results:**

We identified 22 clinical trials (6 randomized and 16 cohort) including 20,199 patients with PCa. Our meta-analysis showed no significant differences in body mass index (SMD = 0.10, 95% CI: −0.08–0.29, p = 0.274) and biopsy Gleason score (GS) (OR = 1.33, 95% CI: 0.76–2.35 p = 0.321) between the two groups. However, the NHT group had a higher mean age (SMD = 0.19, 95% CI: 0.07–0.31, p = 0.001), preoperative prostate-specific antigen (OR = 0.47, 95% CI: 0.19–0.75, p = 0.001), and clinic tumor stage (OR = 2.24, 95% CI: 1.53–3.29, p < 0.001). Compared to the RP group, the NHT group had lower positive surgical margins (PSMs) rate (OR = 0.44, 95% CI: 0.29–0.67, p < 0.001) and biochemical recurrence (BCR) rate (OR = 0.47, 95% CI: 0.26–0.83, p = 0.009). Between both groups, there were no significant differences in estimated blood loss (SMD = −0.06, 95% CI: −0.24–0.13, p = 0.556), operation time (SMD = 0.20, 95% CI: −0.12–0.51, p = 0.219), pathological tumor stage (OR = 0.76, 95% CI: 0.54–1.06, p = 0.104), specimen GS (OR = 0.91, 95% CI: 0.49–1.68, p = 0.756), and lymph node involvement (OR = 0.76, 95% CI: 0.40–1.45, p = 0.404).

**Conclusions:**

NHT prior to RP appeared to reduce the tumor stage, PSMs rate, and risk of BCR in patients with PCa. According to our data, NHT may be more suitable for older patients with higher tumor stage. Besides, NHT may not increase the surgical difficulty of RP.

## Introduction

Prostate cancer (PCa), which generated from an androgen-dependent tumor cell, is the most common malignancy in men worldwide (1). PCa therapy aims to block androgen-dependent growth of tumor cells (2, 3). Theoretically, neoadjuvant hormone therapy (NHT) could offer treatment benefits not only for reducing the size of the prostate volume but also eliminating the invasion of tumor microenvironment (4, 5). NHT before radical prostatectomy (RP) has been reported in several trials involving patients with locally advanced PCa. In several non-randomized trials, NHT plus RP have demonstrated improvements in local control of PCa (6–9).

To date, the use of NHT prior to RP has been explored in multiple studies. The current European Association of Urology guidelines recommend the use of NHT only for patients with intermediate or high-risk PCa if they receive radiation therapy (10). However, due to the lack of solid evidence in survival benefit, the current international guidelines lack clear recommendations for the use of NHT before RP. Some studies have shown favorable effects with respect to tumor stage reduction and decline in rates of positive surgical margins (PSMs), seminal vesicle invasion, and lymph node involvement (LNI) in patients with PCa who received NHT followed by RP (11, 12). However, in numerous trials, the use of NHT before RP has failed to show a definitive benefit in terms of biochemical recurrence (BCR), overall survival (OS), or cancer-specific survival (CSS) (13–15). The aim of the present study was to assess the pathological outcomes of patients who received NHT prior to RP. We attempted to identify which populations of patients with PCa might benefit from NHT and whether NHT might increase the surgical difficulty of RP.

## Materials and Methods

### Search Strategy

A systematic review was performed in accordance with the Preferred Reporting Items for Systematic Reviews and Meta-Analyses (PRISMA) (16). A literature search was performed to identify relevant studies comparing the surgical and oncological outcomes of NHT using PubMed, Web of Science, Chinese National Knowledge Infrastructure (CNKI), and Wanfang databases. The search terms were [prostate cancer] AND [radical prostatectomy] AND [neoadjuvant hormone therapy] AND [surgical outcomes] OR [oncological outcomes]. The search language was restricted to English and Chinese. The reference lists from the retrieved articles were manually searched to identify additional studies. The latest date of this search was July 12, 2020. Ethical approval was not required because we did not conduct clinical research in this meta-analysis.

### Inclusion and Exclusion Criteria

Our search was limited to randomized or observational controlled studies published as full papers. According to the PRISMA guidelines, the selected studies fulfilled the following criteria: (1) literature comparing NHT with non-NHT before RP in patients with pathologically confirmed PCa; (2) randomized controlled trials (RCTs) or retrospective comparative studies in English or Chinese with full text available; (3) evaluation of at least one of the surgical or oncological outcomes; and (4) sufficient data provided for comparison. Exclusion criteria were as follows: (1) non-human research; (2) editorial, case report, review, meta-analysis, and conference abstract; (3) studies that did not analyze patients with PCa; and (4) studies that could not obtain sufficient data to estimate odds ratios (ORs)/standard mean differences (SMDs) and 95% confidence intervals (CIs). When studies were reported on the same population and by the same authors, the study that was well designed and reported more relevant information was used.

### Data Extraction and Study Quality

According to the inclusion criteria, two investigators (HZ and ZZ) independently extracted available data from the eligible studies, and discrepancies were resolved by discussion with a third reviewer (BW). All of the extracted information was recorded according to standardized protocol. The extracted elements included year of publication, first author’s name, study’s country of origin, study design, number of patients, mean age, surgical outcomes (estimated blood loss (EBL) and operation time [OT]), histopathological information (tumor staging, pathological grading, lymph nodes, and surgical margin status), and follow-up data (median/mean follow-up duration, BCR risk).

The methodological quality of RCTs was calculated using the Cochrane risk-of-bias tool (17), which consists of five domains: random sequence generation, allocation concealment, blinding of participants and personnel, incomplete outcome data, and selective reporting. The methodological quality of cohort studies was assessed using the Newcastle Ottawa Scale (NOS) (18). The quality assessment of NOS was evaluated using three broad domains: patient selection, comparability of the study groups, and assessment of outcomes. A score of 0 to 9 was allocated to each study. Studies that received a score of >6 stars were considered to be of high quality.

### Statistical Analysis

The continuous outcome variables were pooled as SMDs with 95% CIs, and the dichotomous variables were pooled as ORs with 95% CIs. Statistical heterogeneity across included studies was calculated by the chi-square-based Q test and *I^2^*. A p < 0.10 or an *I^2^* > 50% was considered as significant between-study heterogeneity. When significant heterogeneity was found in this meta-analysis, the pooled effect was calculated using a random Der-Simonian and Laird effects model; otherwise, the fixed Mantel–Haenszel effects model was used. We performed a subgroup analysis to explore clinical heterogeneity: geographic region (Asia *vs.* non-Asia), year of publication (≥2014 *vs.* <2014), number of patients (≥200 *vs.* <200), and study design (prospective vs. retrospective). Publication bias was evaluated by visual inspection of funnel plots, and Begg’s test was used to further assess publication bias. To explore the stability of our results, a sensitivity analysis was performed by removing one study at a time. All p values were two-tailed, and p < 0.05 was considered as statistically significant. A statistical analysis was conducted using Review Manager Version 5.3 (The Cochrane Collaboration, Oxford, London, UK) and Stata Version 12.0 software (Stata Corp, College Station, TX, USA).

## Results

### Search Results

**Figure 1:**
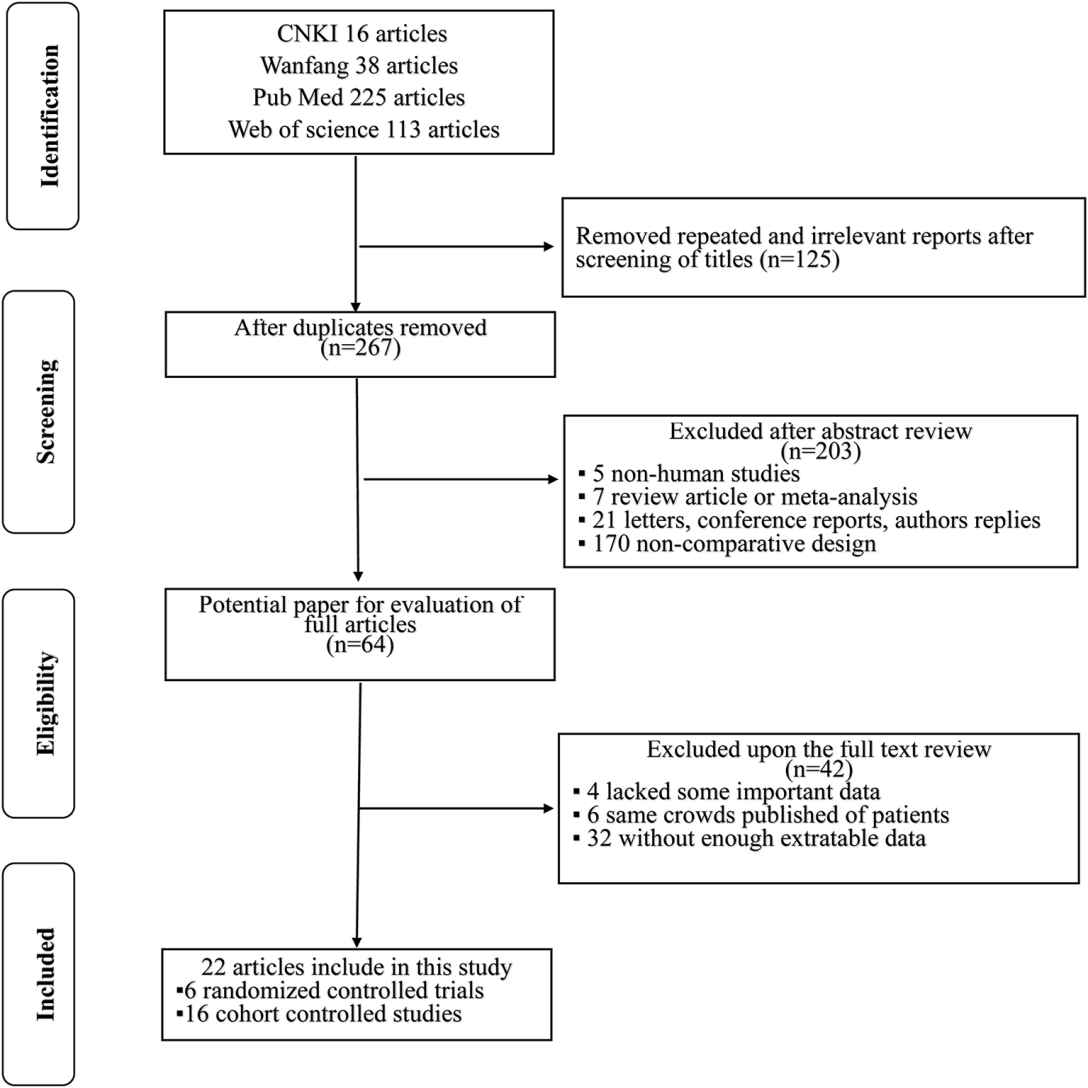
The flowchart of this meta-analysis in accordance with PRISMA guidelines.

Altogether, 392 literature searches were initially identified employing the described search strategy. After screening the titles and abstracts, 328 articles were excluded for various reasons such as repeated and irrelevant reports, non-human studies, letters, case reports, conference reports, reviews, and non-comparative design studies. The remaining 64 articles were assessed in full text. Of those, 42 articles were excluded: 4 lacked key information; 6 duplicated cohorts; and 32 lacked sufficient extractable data. Finally, in accordance with the inclusion criteria, a total of 22 studies (4, 8, 11, 14, 15, 19–35) [6 were RCTs (4, 14, 19, 30, 33, 35) and 16 were retrospective non-randomized studies (8, 11, 15, 20–29, 31, 32, 34)] published from 1996 to 2020 were included in this meta-analysis. A flowchart of the study selection process is shown in **Figure 1**.

### Characteristics of Included Studies

The baseline clinical and pathological characteristics of included studies are shown in **Table 1**. Our data revealed 20,199 patients with PCa (range: 31–14,575). In this study, 3,416 patients with PCa were treated with NHT before RP, whereas 16,783 patients with PCa were treated only with RP. The median or mean age of patients ranged from 61 to 69 years, and the median follow-up duration ranged from 12 months to 99.6 months. Among the studies, eight studies originated from Japan, six were from China, three were from multi-centers, two were from United States, one was from Germany, one was from Canada, and one was from Korea. Various pathological data were collected to compare the oncologic outcomes for the NHT and RP groups (**Table 2**).

**Table 1:** Main characteristics of the eligible studies.

Author	Year	Country	Study design	Recruitment period	No. of patients		Age (years)		p-PSA level(ng.ml-1)		Follow-up time (months)	
					NHT+RP	RP	NHT+RP	RP	NHT+RP	RP	NHT+RP	RP
Chen et al. (19)	2020	Multicenter	Prospective	2011–2013	51	153	Mean ± SD 63.1 ± 8.7	Mean ± SD 63.0 ± 8.1	Mean 16.4	Mean 8.5	24	24
Pan et al. (4)	2019	China	Prospective	2014–2017	73	44	Median (range) 68 (56–78)	Median (range) 69 (57–78)	Median (range) 71.2 (9.7–289)	Median (range) 60.3 (56–78)	Median 18.3	Median 22.8
Kim et al. (11)	2018	Korea	Retrospective	2004–2015	79	97	NA	NA	NA	NA	Mean (range) 49.1 (7.1–148.3)	Mean (range) 49.1 (7.1–148.3)
Tosco et al. (20)	2017	Multicenter	Retrospective	1985–2015	403	1,170	median (IQR) 67 (62–71)	median (IQR) 66 (61–70)	median (IQR) 11 (7–25)	median (IQR) 14 (7–28)	median (IQR) 56 (29–88)	median (IQR) 56 (29–88)
Narita et al. (8)	2017	Japan	Retrospective	2000–2014	280	238	median (IQR) 69 (64–72)	median (IQR) 68 (64–72)	median (IQR) 10.4 (6.9–21.9)	median (IQR) 10.3 (6.6–21.0)	median (IQR) 61 (42–84)	median (IQR) 44(27–60)
Miyata et al. (21)	2017	Japan	Retrospective	NA	73	80	NA	NA	Mean ± SD 14.4 ± 9.8	Mean ± SD 11.9 ± 7.7	NA	NA
Matsumoto et al. (22)	2017	Japan	Retrospective	1996–2017	403	237	median (IQR) 68 (64–72)	median (IQR) 68 (64–72)	median (IQR) 10.1 (6.3–21.2)	median (IQR) 10.3 (6.7–21.1)	median (IQR) 58 (38–84)	median (IQR) 45 (28–61)
Hu et al. (23)	2017	China	Retrospective	2008–2014	24	24	median (IQR) 66 (59–74)	median (IQR) 67 (63–71)	median (IQR) 36.4 (24.8–77.8)	median (IQR) 44.3 (25.6–72.3)	Median 18.5	Median 44.5
Williams et al. (24)	2016	USA	Retrospective	2006–2010	66	149	Mean (range) 61(45-80)	Mean (range) 62 (43-74)	Mean (range) 1.3 (0.1–18.1)	Mean (range) 7.1 (0.7–119)	NA	NA
Koie et al. (25)	2015	Japan	Retrospective	2000–2011	210	210	median (IQR) 68 (64–72)	median (IQR) 68 (62–74)	median (IQR) 9.2 (6.2–17.8)	median (IQR) 8.6 (5.9–16.3)	median (IQR) 37 (18–61)	median (IQR) 52 (33–73)
Takeda et al. (26)	2014	Japan	Retrospective	2006–2011	80	206	Mean ± SD 65.6 ± 6.3	Mean ± SD 64.1 ± 6.5	Mean ± SD 12.7 ± 10.7	Mean ± SD 7.2 ± 3.2	NA	NA
Stewart et al. (27)	2014	USA	Retrospective	1987–2009	1,148	13,427	median (IQR) 62 (56–67)	median (IQR) 63 (57–67)	median (IQR) 7.3 (4.9–13.2)	median (IQR) 6.0 (4.3–9.0)	median (IQR) 99.6 (60–129.6)	median (IQR) 92.4 (46.8–148.8)
Yamamichi et al. (28)	2013	Japan	Retrospective	2007–2010	19	34	median (IQR) 68 (55–75)	median (IQR) 67.5 (51–79)	median (IQR) 9.9 (4.9–67.1)	median (IQR) 6.5 (1.6–19.4)	NA	NA
Naiki et al. (29)	2012	Japan	Retrospective	2004–2009	72	270	Mean ± SD 67.7 ± 5.4	Mean ± SD 66.3 ± 6.1	Mean ± SD 9.8 ± 4.1	Mean ± SD 8.6 ± 5.2	NA	NA
Yang et al. (15)	2011	China	Retrospective	2006–2009	38	31	Mean ± SD 67.3 ± 5.7	Mean ± SD 65.3 ± 5.8	Mean ± SD 14.8 ± 8.1	Mean ± SD 11.4 ± 8.9	NA	NA
Zhou et al. (30)	2009	China	Prospective	2001–2008	26	26	Mean ± SD 65.8 ± 0.9	Mean ± SD 66.5 ± 0.9	Mean ± SD 28.1 ± 26.2	Mean ± SD 18.4 ± 16.9	Mean ± SD 29.4 ± 2.9	Mean ± SD 31.4 ± 4.2
Gao et al. (31)	2009	China	Retrospective	1999–2003	12	19	Mean (range) 61.3 (53–71)	Mean (range) 61.3 (53–71)	NA	NA	NA	NA
Pu et al. (32)	2007	China	Retrospective	2001–2006	44	11	Mean ± SD 63.8 ± 4.4	Mean ± SD 62.9 ± 4.7	Mean ± SD 5.9 ± 0.7	Mean ± SD 5.9 ± 0.8	NA	NA
Maldonado et al. (33)	2006	Germany	Prospective	1999–2005	50	50	Mean ± SD 64 ± 6.2	Mean ± SD 63.7 ± 4.5	Mean ± SD 10.7 ± 10.5	Mean ± SD 8.8 ± 5.3	NA	NA
Namiki et al. (34)	2005	Japan	Retrospective	2000–2002	26	72	Mean ± SD 68.3 ± 5.1	Mean ± SD 66.8 ± 5.1	Mean ± SD 16.9 ± 13.2	Mean ± SD 7.5 ± 2.9	12	12
Soloway et al. (14)	2002	Multicenter	Prospective	1992–1994	138	144	Mean 64.9	Mean 65.4	Mean (range) 14.3 (0.6–50.3)	Mean (range) 12.5 (0.7–54.8)	60	60
Goledberg et al. (35)	1996	Canada	Prospective	1993–1994	101	91	Mean ± SD 62.5 ± 6.0	Mean ± SD 62.2 ± 5.9	NA	NA	NA	NA

NHT, neoadjuvant hormone therapy; RP, radical prostatectomy; SD, standard deviation; IQR, Investment Quality Ranking; NA, data not applicable.

**Table 2:** Tumor characteristics of the eligible studies.

Author	Staging and Grading system	Cycles of NHT	BiopsyGS <7/≥7		SpecimenGS <7/≥7		pT stage1–2/3–4		Clinic stage1–2/3–4		Lymph nodesstatus (positive)		Surgical marginstatus (positive)	
			NHT+RP	RP	NHT+RP	RP	NHT+RP	RP	NHT+RP	RP	NHT+RP	RP	NHT+RP	RP
Chen et al. (19)	NA	6	6/42^*^	18/126^*^	NA	NA	13/17^*^	47/37*	NA	NA	49/2	147/6	23/7*	61/28*
Pan et al. (4)	NA	5	5/68	5/39	NA	NA	31/39	2/42	7/66	6/38	57/13	35/9	57/16	30/14
Kim et al. (11)	2005 ISUP	NA	10/69	16/81	NA	NA	NA	NA	3/76	1/96	57/22	90/7	63/16	61/36
Tosco et al. (20)	NA	NA	113/281^*^	283/854^*^	235/141^*^	669/435^*^	151/252^*^	316/848^*^	113/290^*^	283/887^*^	283/119^*^	743/243^*^	234/168^*^	759/410^*^
Narita et al. (8)	2002 AJCC 2005 ISUP	6	5/276	15/223	NA	NA	172/108	108/130	96/184	56/182	276/4	211/27	254/26	137/101
Miyata et al. (21)	NA	7	22/51	28/52	NA	NA	50/23	48/32	11/62	6/74	68/5	79/1	38/35	38/42
Matsumoto et al. (22)	2009 UICC 2005 ISUP	6	394/9	229/8	394/9	229/8	274/129	108/129	155/248	56/181	399/4	207/30	373/30	135/102
Hu et al. (23)	NA	2-12	2/22	1/23	2/22	0/24	6/18	4/20	17/7	11/13	NA	NA	15/9	2/22
Williams et al. (24)	2002 AJCC	NA	0/66	3/146	0/66	3/146	35/31	62/87	25/41	14/135	NA	NA	44/22	113/36
Koie et al. (25)	2005 ISUP	3	5/205	26/184	NA	NA	115/95	114/96	59/151	43/167	209/1	192/18	194/16	143/67
Takeda et al. (26)	NA	1-14	18/62	83/123	NA	NA	NA	NA	NA	NA	NA	NA	NA	NA
Stewart et al. (27)	2009 AJCC 2005 ISUP	3	501/647	9,455/2,972	433/678^*^	8,065/5,280^*^	797/251^*^	10,634/2,291^*^	144/1,004	484/12,934	1,061/87	12,953/474	903/245	9,896/3,531
Yamamichi et al. (28)	NA	3	10/9	16/19	10/9	7/27	17/3	31/3	NA	NA	NA	NA	13/6	25/9
Naiki et al. (29)	2002 AJCC	4	30/42	121/149	NA	NA	62/10	193/77	NA	NA	NA	NA	52/20	156/114
Yang et al. (15)	2009 AJCC	3	NA	NA	NA	NA	26/12	25/6	NA	NA	NA	NA	30/8	23/8
Zhou et al. (30)	NA	6	NA	NA	NA	NA	19/7	19/7	14/12	4/23	NA	NA	NA	NA
Gao et al. (31)	NA	3-8	NA	NA	NA	NA	NA	NA	NA	NA	1/11	3/16	2/10	7/12
Pu et al. (32)	2002 AJCC	3-8	NA	NA	NA	NA	21/13	8/3	6/38	1/10	NA	NA	39/5	6/5
Maldonado et al. (33)	2002 AJCC	3-9	NA	NA	NA	NA			15/35	32/18	32/18	NA	41/9	42/8
Namiki et al. (34)	NA	3	NA	NA	15/11	34/38	22/4	60/12	3/23	3/69	26/0	72/0	NA	NA
Soloway et al. (14)	NA	3	NA	NA	NA	NA	NA	NA	NA	NA	129/9	136/8	114/24	79/65
Goledberg et al. (35)	NA	3	NA	NA	84/15	80/11	NA	NA	NA	NA	98/3	84/7	73/28	32/59

UICC, Union for International Cancer Control; AJCC, American Joint Committee on Cancer; ISUP, International Society of Urological Pathology; GS, Gleason score; NHT, neoadjuvant hormone therapy; RP, radical prostatectomy; NA, data not applicable.

*means some data missing in the raw data.

### Methodological Quality Assessment

The quality assessment for the six RCTs included in this meta-analysis is shown in **Supplementary Figure S1**. According to the Cochrane risk-of-bias tool, all of the trials were rated with low risk of bias. For the 16 retrospective observational studies, we assessed the quality following the NOS guidelines. The quality of the studies varied from a score of 6 to 8, with a mean of 6 (**Supplementary Table S1**). Therefore, all 22 studies were included in the subsequent analysis.

### Meta-Analysis of Perioperative Variables

**Figure 2:**
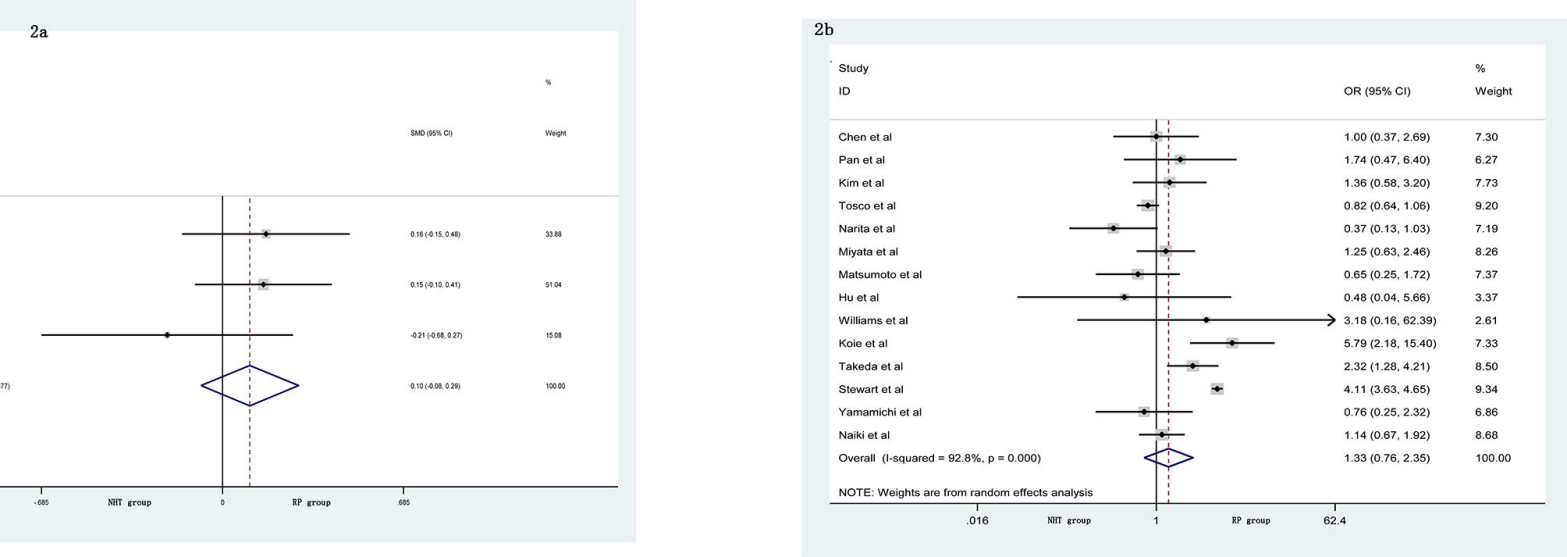
Forest plot of pooled hazard ratios for perioperative variables: **(A)** BMI; **(B)** biopsy GS. No significant differences between the NHT and RP groups were found.

**Figure 3:**
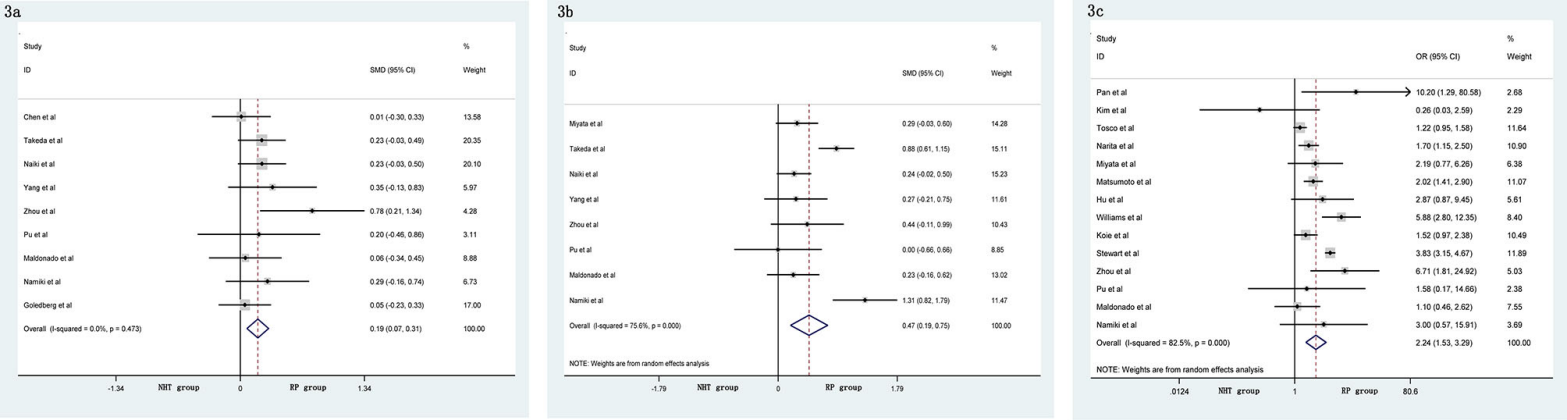
Forest plot of pooled hazard ratios for perioperative variables, NHT group had a higher: **(A)** advanced age; **(B)** p-PSA; **(C)** clinic tumor stage than RP group.

The pooled data from the included studies that reported body mass index (BMI) (SMD = 0.10, 95% CI: −0.08–0.29, p = 0.274, **Figure 2A**) and biopsy Gleason score (GS) (OR = 1.33, 95% CI: 0.76–2.35, p = 0.321, **Figure 2B**) showed no significant differences between the NHT and RP groups. However, the NHT group had a higher mean age (SMD = 0.19, 95% CI: 0.07–0.31, p = 0.001, **Figure 3A**), preoperative prostate-specific antigen (p-PSA) (OR = 0.47, 95% CI: 0.19–0.75, p = 0.001, **Figure 3B**), and clinic tumor stage (OR = 2.24, 95% CI: 1.53–3.29, p < 0.001, **Figure 3C**).

### Meta-Analysis of Postoperative Variables

**Figure 4:**
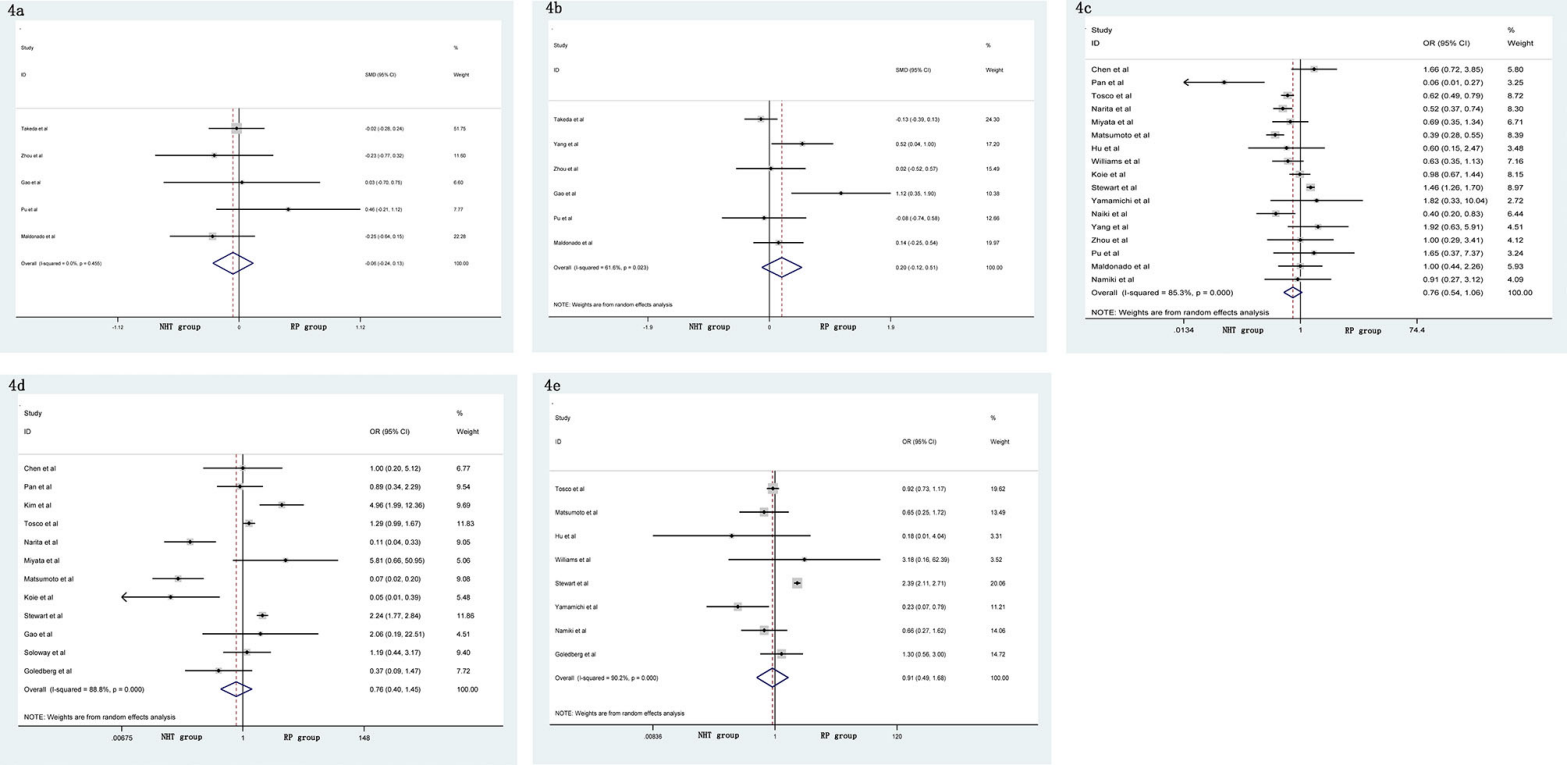
Forest plot of pooled hazard ratios for postoperative variables: **(A)** EBL; **(B)** OT; **(C)** pathological tumor stage; **(D)** LNI; **(E)** specimen GS. No significant differences between the NHT and RP groups were found.

**Figure 5:**
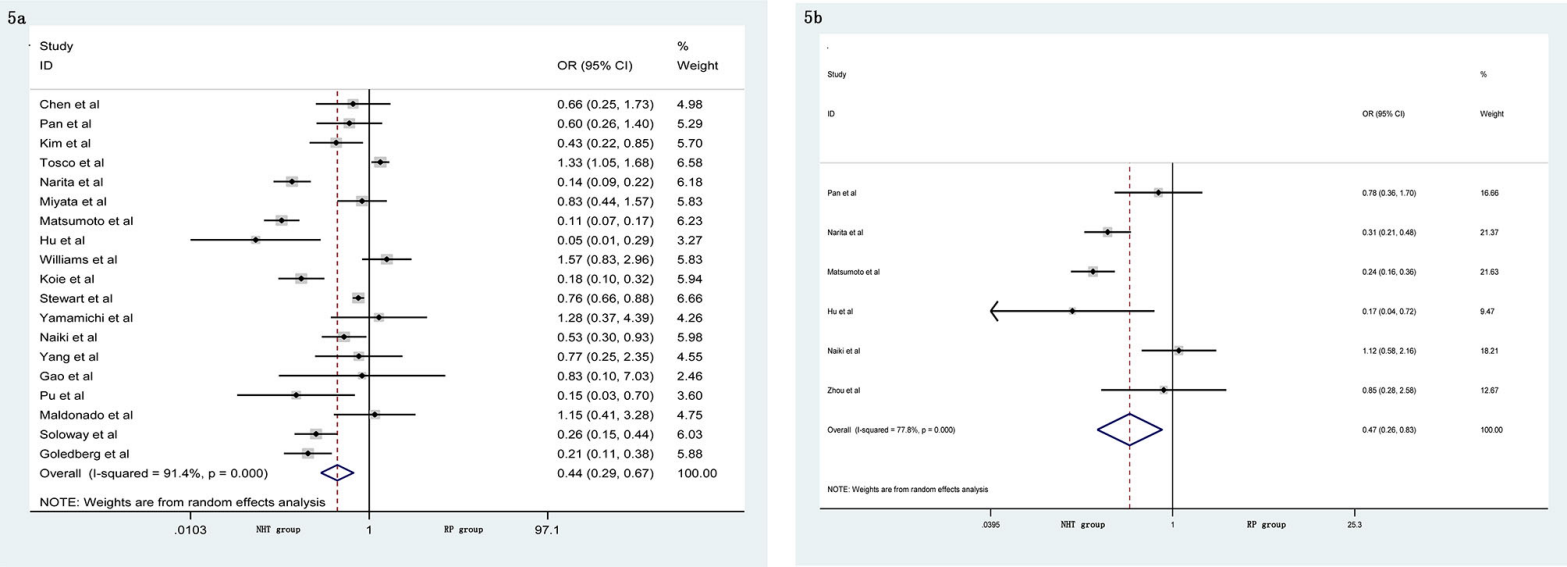
Forest plot of pooled hazard ratios for postoperative variables, NHT group had a lower **(A)** PSMs; **(B)** BCR than RP group.

A meta-analysis did not show significant differences in EBL (SMD = −0.06, 95% CI: −0.24–0.13, p = 0.556, **Figure 4A**), OT (SMD = 0.20, 95% CI: −0.12–0.51, p = 0.219, **Figure 4B**), pathological tumor stage (OR = 0.76, 95% CI: 0.54–1.06, p = 0.104, **Figure 4C**), LNI (OR = 0.76, 95% CI: 0.40–1.45, p = 0.404, **Figure 4D**), and specimen GS (OR = 0.91, 95% CI: 0.49–1.68, p = 0.756, **Figure 4E**) between the NHT and RP groups. Compared with the RP group, the NHT group had lower PSMs rate (OR = 0.44, 95% CI: 0.29–0.67, p < 0.001, **Figure 5A**) and BCR risk (OR = 0.47, 95% CI: 0.26–0.83, p = 0.009, **Figure 5B**).

### Subgroup Analysis

To discover the source of heterogeneity, a subgroup analysis was applied with a random effects model. Considering the potential factors, we performed a subgroup analysis by geographic region, year of publication, number of patients, and study design. Because there was no significant heterogeneity detected for advanced age, BMI, and EBL, a subgroup analysis was not performed. For the perioperative variables, subgroup analyses showed that the NHT group had significantly higher p-PSA and clinic tumor stage in the prospective study compared to the RP group (**Table 3**). For the postoperative variables, no significant changes occurred in the subgroup analyses for OT, pathological tumor stage, specimen GS, LNI, and PSMs (**Table 4**). However, the results varied in some subgroup analyses. The reason for the above difference may be related to the small number of studies included in the subgroup analysis.

**Table 3:** Summary and subgroup analysis of preoperative clinicopathological features for the PCa patients treated with NHT before RP.

Analysis specification	No. of studies	Study heterogeneity		Effects model	Pooled OR/SMD (95% CI)	*P*-Value
		*I* ^2^ (%) *P_heterogeneity_*				
**Mean age**						
Overall	9	0	0.473	Fixed	0.19(0.07,0.31)	0.001
**BMI**						
Overall	3	0	0.377	Fixed	0.10(−0.08,0.29)	0.274
**p-PSA**						
Overall	8	75.6	<0.001	Random	0.47(0.19,0.75)	0.001
Geographical region						
Asia	7	77.8	<0.001	Random	0.50(0.19,0.81)	0.002
Non-Asia	1	–	–	–	–	–
Year of publication						
≥2014	2	87.1	0.005	Random	0.59(0.01,1.17)	0.047
<2014	6	71	0.004	Random	0.42(0.08,0.75)	0.015
No. of patients						
≥200	2	91.0	0.001	Random	0.56(−0.07,1.18)	0.080
<200	6	69.5	0.006	Random	0.43(0.09,0.77)	0.014
Study design						
Prospective	2	0	0.540	Fixed	0.30(−0.02,0.62)	0.005
Retrospective	6	81.4	<0.001	Random	0.51(0.16,0.86)	0.066
**Clinic T-stage**						
Overall	14	82.5	<0.001	Random	2.24(1.53,3.29)	<0.001
Geographical region						
Asia	10	19.8	0.261	Fixed	1.95(1.48,2.57)	<0.001
Non-Asia	4	94.9	<0.001	Random	2.36(1.05,5.32)	0.039
Year of publication						
≥2014	10	86.9	<0.001	Random	2.23(1.45,3.43)	<0.001
<2014	4	44.1	0.147	Fixed	2.32(0.91,5.93)	0.079
No. of patients						
≥200	5	85.8	<0.001	Random	1.41(1.17,1.69)	<0.001
<200	4	42.9	0.154	Fixed	1.38(1.20,1.58)	<0.001
Study design						
Prospective	3	73.3	0.023	Random	3.58(0.79,16.11)	0.097
Retrospective	11	85.1	<0.001	Random	2.13(1.41,3.22)	<0.001
**Biopsy GS**						
Overall	14	92.8	<0.001	Random	1.33(0.76,2.35)	0.321
Geographical region						
Asia	10	60.2	0.007	Random	1.26(0.81,1.97)	0.311
non-Asia	4	97.7	<0.001	Random	1.67(0.49,5.65)	0.410
Year of publication						
≥2014	12	93.4	<0.001	Random	1.42(0.75,2.66)	0.280
<2014	2	0	0.521	Fixed	1.06(0.66,1.70)	0.821
No. of patients						
≥200	9	95.2	<0.001	Random	1.46(0.71,3.00)	0.308
<200	5	0	0.810	Fixed	1.19(0.77,1.86)	0.438
Study design						
Prospective	2	0	0.505	Fixed	1.33(0.56,2.69)	0.612
Retrospective	12	93.8	<0.001	Random	1.34(0.72,2.48)	0.357

**Table 4:** Summary and subgroup analysis of postoperative clinicopathological features for the PCa patients treated with NHT before RP.

Analysis specification	No. of studies	Study heterogeneity		Effects model	Pooled OR/SMD (95% CI)	*P*-Value
		*I^2^* (%) *P_heterogeneity_*				
**EBL**						
Overall	5	0	0.455	Fixed	−0.06(−0.24,0.13)	0.556
**OT**						
Overall	6	61.6	0.023	Random	0.20(−0.12,0.51)	0.219
Geographical region						
Asia	5	69	0.012	Random	0.23(−0.18,0.63)	0.269
Non-Asia	1	–	–	–	–	–
Year of publication						
≥2014	1	–	–	–	–	–
<2014	5	49.4	0.095	Random	0.30(−0.05,0.64)	0.089
No. of patients						
≥200	1	–	–	–	–	–
<200	5	49.4	0.095	Random	0.30(−0.05,0.64)	0.089
Study design						
Prospective	2	0	0.727	Fixed	0.10(−0.22,0.42)	0.527
Retrospective	4	76.7	0.005	Random	0.30(−0.23,0.82)	0.265
**Pathological T-stage**						
Overall	17	85.3	<0.001	Random	0.76(0.54,1.06)	0.104
Geographical region						
Asia	12	65.9	0.001	Random	0.65(0.45,0.94)	0.021
Non-Asia	5	90	<0.001	Random	0.97(0.58,1.63)	0.906
Year of publication						
≥2014	10	91.1	<0.001	Random	0.66(0.44,1.01)	0.057
<2014	7	24.2	0.244	Fixed	0.94(0.58,1.50)	0.786
No. of patients						
≥200	8	92.4	<0.001	Random	0.72(0.47,1.11)	0.140
<200	9	53.1	0.029	Random	0.82(0.46,1.44)	0.486
Study design						
Prospective	4	81.1	0.001	Random	0.63(0.19,2.10)	0.147
Retrospective	13	87.2	<0.001	Random	0.76(0.53,1.10)	0.454
**Specimen GS**						
Overall	8	90.2	<0.001	Random	0.91(0.49,1.68)	0.756
Geographical region						
Asia	5	32.5	0.205	Fixed	0.63(0.35,1.16)	0.140
Non-Asia	3	95.8	<0.001	Random	1.58(0.65,3.84)	0.311
Year of publication						
≥2014	5	92.6	<0.001	Random	1.16(0.54,2.51)	0.701
<2014	3	61.5	0.074	Random	0.64(0.26,1.58)	0.332
No. of patients						
≥200	5	93.0	<0.001	Random	1.13(0.58,2.29)	0.740
<200	3	66.2	0.052	Random	0.50(0.12,2.04)	0.331
Study design						
Prospective	1	–	–	–	–	–
Retrospective	7	91.5	<0.001	Random	0.85(0.43,1.68)	0.633
**LNI**						
Overall	12	88.8	<0.001	Random	0.76(0.40,1.45)	0.404
Geographical region						
Asia	8	88.1	<0.001	Random	0.52(0.14,1.97)	0.336
Non-Asia	4	72.2	0.013	Random	1.57(1.00,2.43)	0.056
Year of publication						
≥2014	9	91.5	<0.001	Random	0.73(0.34,1.54)	0.405
<2014	3	15.8	0.305	Fixed	0.87(0.36,2.07)	0.748
No. of patients						
≥200	7	93.0	<0.001	Random	0.48(0.21,1.10)	0.083
<200	5	69.4	0.011	Random	1.68(0.55,5.13)	0.363
Study design						
Prospective	4	0	0.593	Fixed	0.85(0.48,1.52)	0.466
Retrospective	8	92.5	<0.001	Random	0.73(0.32,1.69)	0.590
**PSM**						
Overall	19	91.4	<0.001	Random	0.44(0.29,0.67)	<0.001
Geographical region						
Asia	13	81.8	<0.001	Random	0.32(0.19,0.51)	<0.001
Non-Asia	6	87.2	<0.001	Random	0.82(0.52,1.29)	0.396
Year of publication						
≥2014	11	94.4	<0.001	Random	0.42(0.24,0.73)	0.002
<2014	8	62	0.010	Random	0.46(0.27,0.77)	0.003
No. of patients						
≥200	9	95.6	<0.001	Random	0.41(0.23,0.76)	0.004
<200	10	64.3	0.003	Random	0.48(0.28,0.81)	0.006
Study design						
Prospective	5	67.5	0.015	Random	0.43(0.24,0.78)	0.006
Retrospective	14	93	<0.001	Random	0.44(0.26,0.72)	0.001
**BCR**						
Overall	6	77.8	<0.001	Random	0.47(0.26,0.83)	0.009
Year of publication						
≥2014	4	60.1	0.057	Random	0.33(0.20,0.53)	<0.001
<2014	2	0	0.682	Fixed	1.04(0.59,1.84)	0.892
No. of patients						
≥200	3	87.0	<0.001	Random	0.42(0.20,0.89)	0.024
<200	3	46.5	0.154	Random	0.56(0.24,1.32)	0.185
Study design						
Prospective	2	0	0.895	Fixed	0.80(0.42,1.52)	0.497
Retrospective	4	81.6	0.001	Random	0.37(0.19,0.73)	0.004

### Publication Bias and Sensitivity Analysis

**Figure 6:**
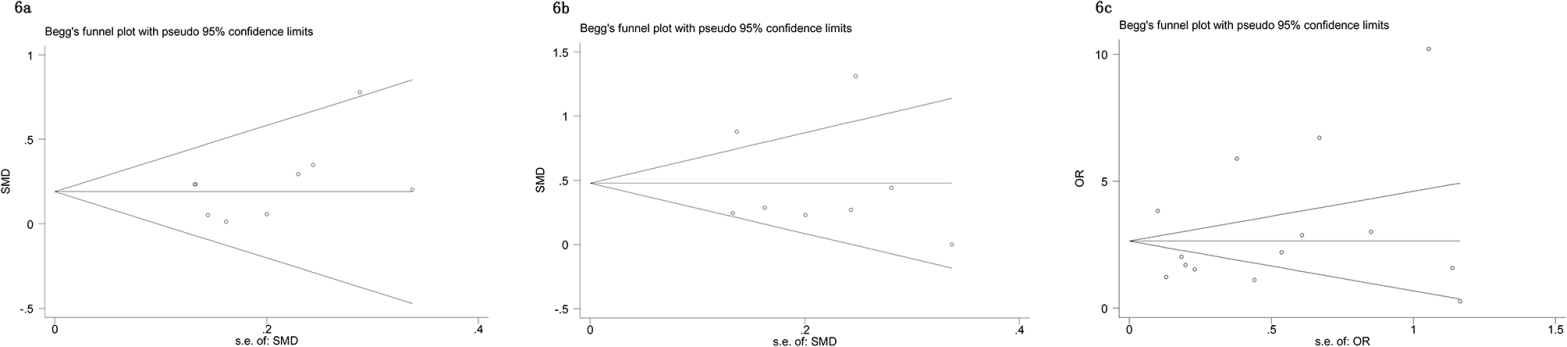
Funnel plots to explore publication bias in the estimates of perioperative variables: **(A)** advanced age; **(B)** p-PSA; **(C)** clinic tumor stage. No significant publication bias was found.

**Figure 7:**
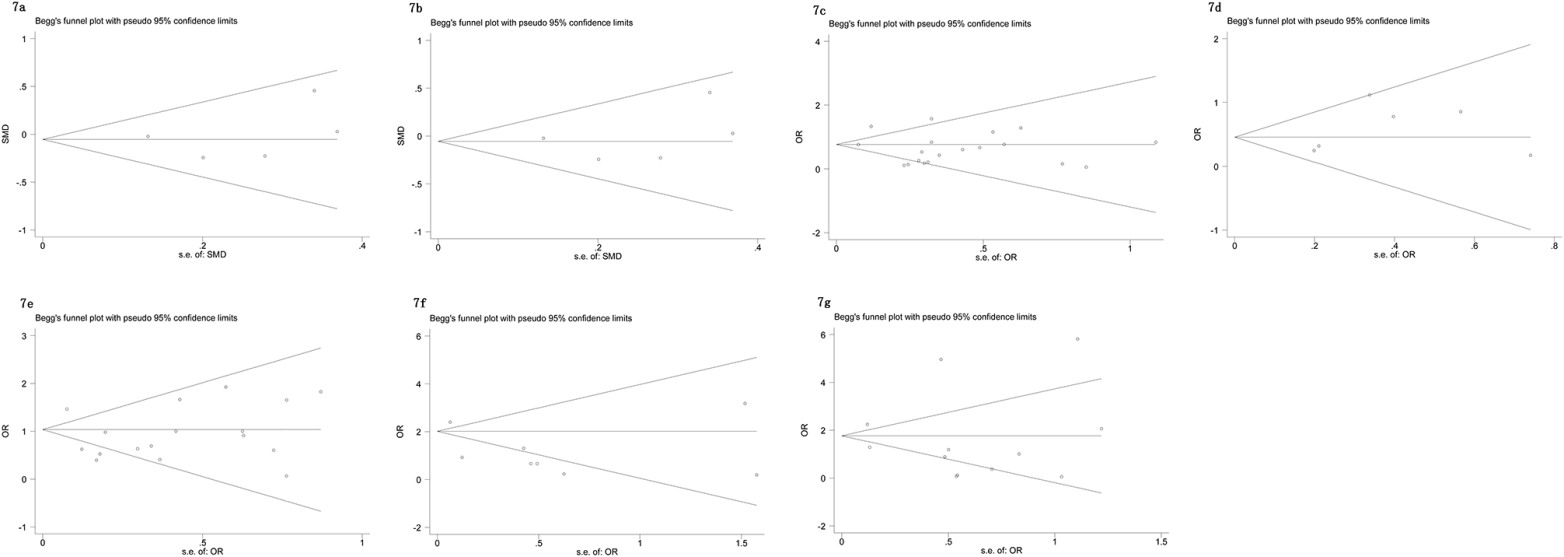
Funnel plots to explore publication bias in the estimates of postoperative variables: **(A)** EBL; **(B)** OT; **(C)** PSMs; **(D)** BCR; **(E)** pathological tumor stage; **(F)** specimen GS; **(G)** LNI. No significant publication bias was found.

The Begg’s tests and funnel plots were adopted to detect potential publication bias in the present meta-analysis. As shown in **Figure 6**, the funnel plots indicated that the included studies had no evident asymmetry. Furthermore, the results from the Begg’s test for the included studies assessing the perioperative variables were as follows: advanced age (p = 0.223, **Figure 6A**), p-PSA (p = 0.873, **Figure 6B**), and clinic tumor stage (p = 0.821, **Figure 6C**); and the postoperative variables were as follows: EBL (p = 0.682, **Figure 7A**), OT (p = 0.129, **Figure 7B**), PSMs (p = 0.241, **Figure 7C**), BCR (p = 0.300, **Figure 7D**), pathological tumor stage (p = 0.315, **Figure 7E**), specimen GS (p = 0.181, **Figure 7F**), and LNI (p = 0.749, **Figure 7G**). Although we found a slight publication bias in biopsy GS (p = 0.047), no significant publication bias was found in the other parameters.

A sensitivity analysis was conducted using the leave-one-out approach to examine the stability of the current study. The selected studies were sequentially omitted to investigate whether any individual study influenced the results (**data not shown**). The results showed no significant changes in our pooled results, suggesting the robustness of the pooled results. Funnel plot and sensitivity analysis for BMI were not conducted because of the small size.

## Discussion

PCa is the most commonly diagnosed malignancy in men and the second most common cause of cancer-related death in the USA (36). Although controversy exists regarding the definitive treatment recommendations (radiation therapy, RP, or expectant management) for localized and high-risk PCa, RP has long remained the preferred therapeutic option for localized PCa (37). The ultimate goal of RP is the complete removal of all cancer cells. Unfortunately, this is not always possible, not even in carefully selected patients. As a result, incomplete resection of PCa cancer cells may result in a higher risk of local or distant metastasis (38). Presently, the systematic treatment of high-risk localized PCa has been the consensus of many clinicians (39, 40).

Given the advances in PCa systemic therapy, much interest centers on the application of NHT. NHT is defined as a systemic therapy that is administered before commencing definitive locoregional therapy (41). The proposed mode of NHT is based on the hypothesis that androgen blockade may induce PCa cell death (apoptosis) by inhibiting the growth of hormone sensitive cells, which will downstage the tumor prior to RP (42). By theoretically increasing the likelihood of organ confined tumors, the efficacy of RP is augmented. Considering these findings, NHT prior to RP has been widely performed.

The reason for using NHT in PCa was to decrease the size of the prostate volume (43). Decreasing prostate size might help the urologist to remove the prostate more efficiently and easily, with fewer intraoperative comorbidities during RP (24). Use of NHT before RP was assessed in different RCTs showing higher rates of downstaging and lower rates of PSMs compared to RP alone (14, 19, 32). In contrast, NHT administered before RP has yet to show a definitive survival benefit. This stems from the numerous trials that have demonstrated a lack of statistically significant improvement in CSS and OS (13, 15, 44). However, why NHT fails to improve survival is unclear. One possible reason is that androgen-resistant tumor cells may exist in early PCa. Another potential reason is that NHT cannot sufficiently suppress androgen levels in prostate tissue, thereby significantly destroying tumor cells. Despite the international guidelines advising against its use (37), the administration of NHT prior to RP remains controversial. Presently, many urologists incorporate NHT and RP in their clinical practice.

No clear evidence shows that NHT is beneficial in patients with PCa. Some studies have indicated that patients treated with NHT have fewer PSMs but without improving BCR after RP. Naiki et al. (29) and Soloway et al. (14) found that pre-surgical NHT is beneficial for PCa control by suppressing BCR. In this study, the efficacy of NHT was statistically significant in reducing the tumor stage after RP, PSMs rates, and BCR risk. However, no significant differences were detected between the two groups regarding biopsy GS, specimen GS, and LNI. This meta-analysis demonstrated that the NHT group comprised patients who were significantly older and were classified at a high clinic tumor stage, which means that NHT tends to be used more often in elderly patients and patients with non-localized PCa. The subgroup analyses of the perioperative variables, however, demonstrated that the NHT group had significantly higher p-PSA and higher clinic tumor stage in the prospective study. We speculate that the reasons for the above differences may come from the study population, pathological backgrounds, and methods of NHT.

The potential surgical advantages of NHT during RP remain a subject of debate. Some researchers have stated that NHT decreases the operative parameters and thereby facilitates the surgical procedure (26, 29, 45). Others have reported no differences in OT, EBL, and complication rate in patients who received or did not receive NHT prior to RP (15, 33). Narita et al. (8) reported that the patients who received NHT required a longer OT and a higher transfusion rate than the non-NHT patients. However, Hu et al. (23) found that the OT was significantly shorter and that the EBL was significantly less in the NHT group. Our data did not show that use of NHT resulted in increased OT or greater EBL, which means that NHT may not increase the surgical difficulty of RP. These results could be useful for surgeons, but additional large-scale RCT studies are warranted to draw more definitive conclusions on NHT.

Several potential limitations in this study should be acknowledged. First, except for six RCTs, all of the studies included were observational. Although we have conducted a quality evaluation of all the included literature, the quality of eligible studies is a concern because the included studies were conducted with different modes and levels of surgical expertise. Second, obvious heterogeneity among studies was identified in several analyses. Different study design, patients’ baseline features, surgical approach, adequacy of follow-up, and measurement of outcomes might be potential contributors to the heterogeneity. Therefore, the random effect model was used to reduce the impact of heterogeneity but could not completely eliminate it. Third, the data included in these documents span a lengthy time period. Therefore, selection bias of the original articles remains a significant limitation in the current study. It is reasonable to believe that the recent technical developments in PCa make the NHT of today more complete than it was 30 years ago. Finally, the follow-up duration was quite short in several included studies, and we did not synthesize the evidence to assess the relative differences in the long-term survival outcomes because of the limited number of studies that provided the data.

Despite these limitations, there are several strengths in our study. Our research was conducted at an appropriate time because this problem urgently needs to be resolved and sufficient clinical research has accumulated in recent decades to permit analysis. In the present study, relying on the PRISMA, we strictly adhered to the established inclusion and exclusion criteria, carefully assessed the quality of the included literature, and further performed subgroup and sensitivity analyses to minimize heterogeneity differences. Therefore, the reliability and the stability of our results are guaranteed.

## Conclusion

This meta-analysis showed that NHT prior to RP appeared to reduce the pathological tumor stage, PSMs rate, and risk of BCR in patients with PCa. Altogether, based on our data, NHT may be more suitable for older patients with high PCa clinical stages. The clinical application of NHT on PCa should carefully consider potential risks and benefits to ensure maximizing treatment benefits. Given the inherent limitations of the included studies, additional well-designed RCTs with proper inclusion and exclusion criteria are required to confirm our findings.

## Data Availability Statement

The original contributions presented in the study are included in the article/**Supplementary Material**. Further inquiries can be directed to the corresponding author.

## Author Contributions

LZ: Project development and manuscript writing. BW: Data management. HZ: Data collection. ZZ: Data collection. JY: Data analysis, data management. YF: Data analysis, data management. All authors contributed to the article and approved the submitted version.

## Conflict of Interest

The authors declare that the research was conducted in the absence of any commercial or financial relationships that could be construed as a potential conflict of interest.

## Supplementary Material

Supplementary Figure 1 Risk of bias graph (up: risk of bias graph; down: risk of bias summary), all of the trials were rated with low risk of bias.

Supplementary Table 1 Quality assessment of cohort studies included in this meta-analysis.
