# Supplementary figures and images for: The Impact of Neoadjuvant Hormone Therapy on Surgical and Oncological Outcomes for Patients With Prostate Cancer Before Radical Prostatectomy: A Systematic Review and Meta-Analysis
Source: Front Oncol. 2021 Feb 8;10:615801. doi: 10.3389/fonc.2020.615801 (PMC7897693; doi:10.3389/fonc.2020.615801)

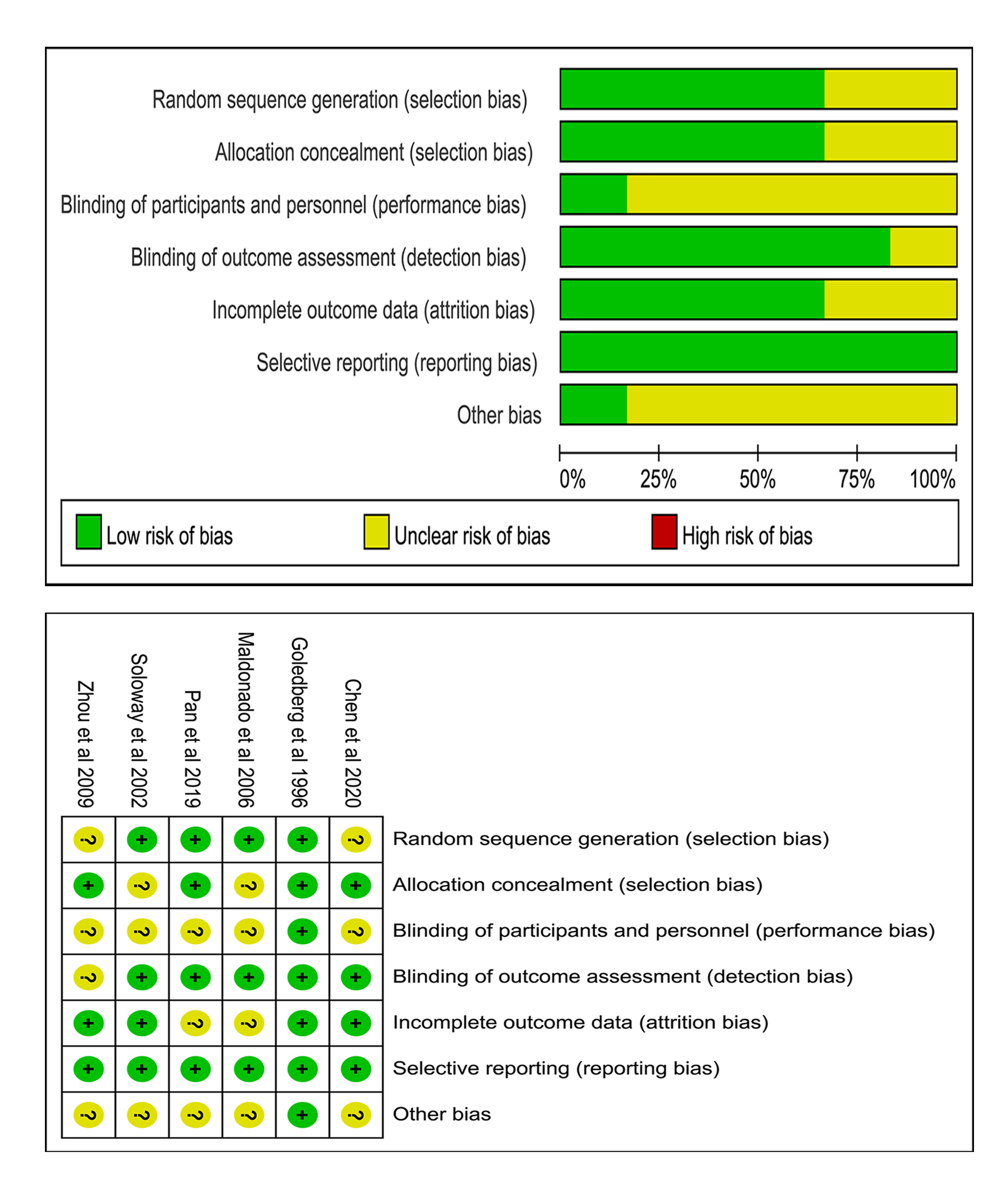

Supplement: Supplementary Figure 1 — Risk of bias graph (up: risk of bias graph; down: risk of bias summary), all of the trials were rated with low risk of bias. [file Image_1.tif]
