# Supplementary material for: The Impact of Neoadjuvant Hormone Therapy on Surgical and Oncological Outcomes for Patients With Prostate Cancer Before Radical Prostatectomy: A Systematic Review and Meta-Analysis
Source: Front Oncol. 2021 Feb 8;10:615801. doi: 10.3389/fonc.2020.615801 (PMC7897693; doi:10.3389/fonc.2020.615801)
Supplement: Supplementary Table 1 — Quality assessment of cohort studies included in this meta-analysis. [file Table_1.docx]

**Supplementary table S1**.Quality assessment of cohort studies included in this meta- analysis

| **Study** | **Representativeness of the exposed cohort** | **Selection of the unexposed cohort** | **Ascertainment of exposure** | **Outcome of interest not present at start of study** | **Control for important factor or additional factor** | **Outcome assessment** | **Follow-up long enough for outcomes to occur** | **Adequacy of follow-up of cohort** | **Total quality scores** |
| --- | --- | --- | --- | --- | --- | --- | --- | --- | --- |
| Kim et al[^11^](#_ENREF_11) | ★ | ★ | ★ | ★ | ★ | ★ | ★ | ★ | 8 |
| Tosco et al[^20^](#_ENREF_20) | ★ | ★ | ★ | ★ | ★ | ★ | ★ | ★ | 8 |
| Narita et al[^8^](#_ENREF_8) | ★ | ★ | ★ | ★ | — | ★ | ★ | ★ | 7 |
| Miyata et al[^21^](#_ENREF_21) | ★ | — | ★ | ★ | ★ | ★ | ★ | — | 6 |
| Matsumoto et al[^22^](#_ENREF_22) | ★ | ★ | ★ | ★ | ★ | ★ | ★ | ★ | 8 |
| Hu et al[^23^](#_ENREF_23) | ★ | — | ★ | ★ | ★ | ★ | ★ | ★ | 7 |
| Williams et al[^24^](#_ENREF_24) | ★ | ★ | ★ | ★ | — | ★ | ★ | — | 6 |
| Koie et al[^25^](#_ENREF_25) | ★ | ★ | ★ | ★ | ★ | ★ | ★ | ★ | 8 |
| Takeda et al[^26^](#_ENREF_26) | ★ | ★ | ★ | ★ | ★ | ★ | ★ | — | 7 |
| Stewart et al[^27^](#_ENREF_27) | ★ | ★ | ★ | ★ | ★ | ★ | ★ | ★ | 8 |
| Yamamichi et al[^28^](#_ENREF_28) | ★ | ★ | ★ | ★ | ★ | ★ | ★ | — | 7 |
| Naiki et al[^29^](#_ENREF_29) | ★ | ★ | ★ | ★ | ★ | ★ | ★ | — | 7 |
| Yang et al[^15^](#_ENREF_15) | ★ | ★ | ★ | ★ | ★ | ★ | ★ | — | 7 |
| Gao et al[^31^](#_ENREF_31) | ★ | — | ★ | ★ | ★ | ★ | ★ | — | 6 |
| Pu et al[^32^](#_ENREF_32) | ★ | ★ | ★ | ★ | ★ | ★ | ★ | — | 7 |
| Namiki et al[^34^](#_ENREF_34) | ★ | ★ | ★ | ★ | — | ★ | ★ | — | 6 |
